# 3-(4-Meth­oxy­benzo­yl)-6-nitro­coumarin

**DOI:** 10.1107/S1600536813002948

**Published:** 2013-02-06

**Authors:** Saleta Vazquez-Rodriguez, Eugenio Uriarte, Lourdes Santana

**Affiliations:** a Department of Organic Chemistry, University of Santiago de Compostela, Santiago de Compostela, A Coruna, Spain

## Abstract

In the title coumarin derivative (also known as 2*H*-chromen-2-one or 2*H*-1-benzopyran-2-one), C_17_H_11_NO_6_, the coumarin ring system is nearly planar, with a dihedral angle of 3.35 (9)° between the pyrone and the benzene rings. The dihedral angle between the planes formed by the coumarin ring system and the benzene substituent is 54.60 (7)°, clearly showing the non-coplanarity of the whole aromatic system. The crystal studied was a non-merohedral twin; the minor component refined to approximately 0.44.

## Related literature

For the synthesis of the title compound, see: Raju *et al.* (2010 ▶). For examples of the biological activity of coumarin derivatives, see: Borges *et al.* (2009 ▶), Matos *et al.* (2011*a* ▶,*b* ▶,*c* ▶), Viña *et al.* (2012*a* ▶,*b* ▶); Vazquez-Rodriguez *et al.* (2013 ▶).
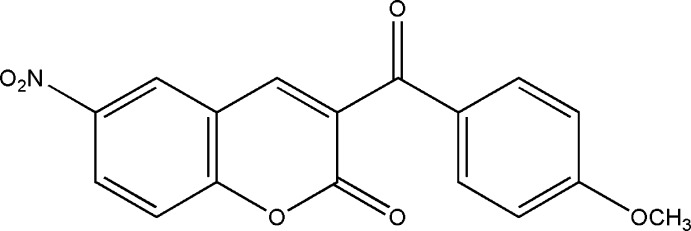


## Experimental

### 

#### Crystal data

C_17_H_11_NO_6_
*M* *_r_* = 325.27
Monoclinic, 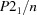

*a* = 8.875 (3) Å
*b* = 17.266 (5) Å
*c* = 9.174 (3) Å
β = 95.401 (15)°
*V* = 1399.6 (7) Å^3^
*Z* = 4
Mo *K*α radiation
μ = 0.12 mm^−1^
*T* = 100 K
0.67 × 0.14 × 0.03 mm

#### Data collection

Bruker APEXII CCD diffractometer
Absorption correction: multi-scan (*SADABS*; Bruker, 2007 ▶) *T*_min_ = 0.604, *T*_max_ = 0.745
30736 measured reflections
2864 independent reflections
2200 reflections with *I* > 2σ(*I*)
*R*_int_ = 0.054

#### Refinement

*R*[*F* ^2^ > 2σ(*F* ^2^)] = 0.043
*wR*(*F* ^2^) = 0.126
*S* = 0.91
2864 reflections
219 parameters
H-atom parameters constrained
Δρ_max_ = 0.39 e Å^−3^
Δρ_min_ = −0.26 e Å^−3^

### 

Data collection: *APEX2* (Bruker, 2007 ▶); cell refinement: *SAINT* (Bruker, 2007 ▶); data reduction: *SAINT*; program(s) used to solve structure: *SIR97* (Altomare *et al.*, 1999 ▶); program(s) used to refine structure: *SHELXL97* (Sheldrick, 2008 ▶); molecular graphics: *ORTEP-3 for Windows* (Farrugia, 2012 ▶); software used to prepare material for publication: *WinGX* (Farrugia, 2012 ▶).

## Supplementary Material

Crystal structure: contains datablock(s) global, I. DOI: 10.1107/S1600536813002948/go2080sup1.cif

Structure factors: contains datablock(s) I. DOI: 10.1107/S1600536813002948/go2080Isup2.hkl

Supplementary material file. DOI: 10.1107/S1600536813002948/go2080Isup3.cml

Additional supplementary materials:  crystallographic information; 3D view; checkCIF report

## Figures and Tables

**Fig. 1.**
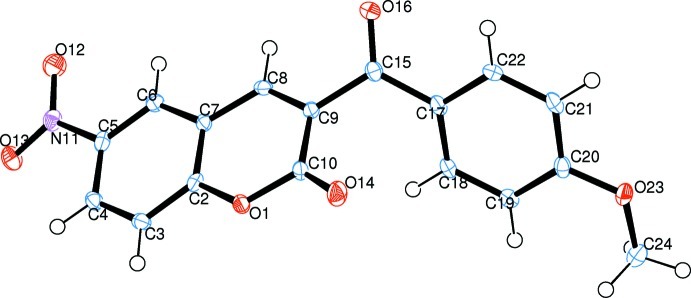
Molecular structure of the title compound with the atom-numbering scheme. Displacement ellipsoids are drawn at the 50% probability level.

**Fig. 2.**
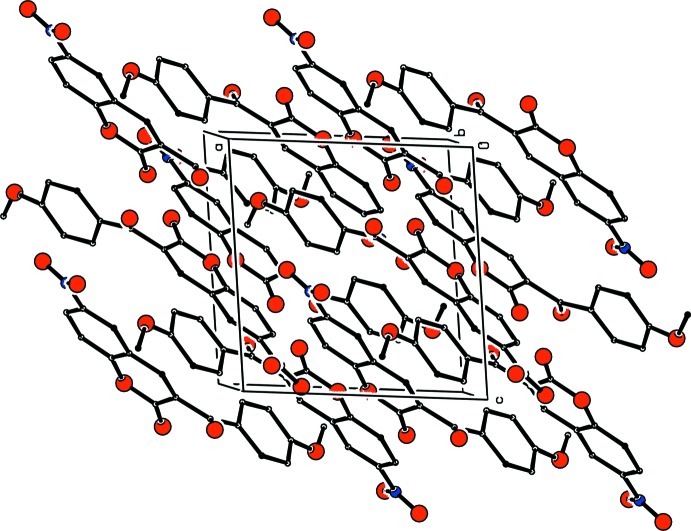
Packing diagram of the title structure viewed along the b axis.

## supplementary crystallographic information

### Comment

Coumarin derivative compounds present a great interest in the medicinal chemistry field due to the displayed biological properties that they present (Borges *et al.* 2009, Matos *et al.* 2011*a*, Matos *et al.* 2011*b*, Matos *et al.* 2011*c*, Vazquez-Rodriguez *et al.* 2013, Viña *et al.* 2012*a* and Viña *et al.* 2012*b*). The title structure is a 3-substituted coumarin derivative containing a 4-methoxybenzoyl ring at the mentioned position and a nitro group at position 6 of the coumarin scaffold. Therefore, the X-ray analysis of this compound (figure 1) aims to contribute to the elucidation of structural requirements needed to understand the partial planarity of the compound (coumarin nucleus) and the torsion of the 3-benzoyl moiety regarding to this nucleus. From the single-crystal diffraction measurements one can conclude that both the pyrone and benzene rings in the coumarin motif are essentially planar, presenting dihedral angle of 3.35 (9)°. The planarity of the coumarin moiety is also evident by the torsion angle value between their carbons C3—C2—C7—C8 (-175.89 (18)°).

In addition, the torsion angles of the carbonyl group *versus* the coumarin moiety and the phenyl ring are C10—C9—C15—O16 (43.2 (2)°) and O16—C15—C17—C18 (-152.9 (2)°) respectively. These values are typical of the torsion permitted by the rotation present at position 3. Presence of the carbonyl group at position 3 provokes a non coplanarity of the benzoyl moiety regarding to the coumarin scaffold. This fact is evident taking into account the dihedral angles formed by the planes of the coumarin, the carbonyl and the phenyl groups. Dihedral angle between the coumarin moiety and the carbonyl group is 38.66 (9)°; between the carbonyl and the phenyl group is 25.76 (10)° and between the coumarin scaffold and the phenyl group is 54.60 (7)°.

### Experimental

*3-(4-Methoxybenzoyl)-6-nitrocoumarin* was prepared according to the following protocol: to a solution of 2-hydroxy-5-nitrobenzaldehyde (1 mmol) and ethyl 4-methoxybenzoylacetate (1 mmol) in ethanol (4 ml), a catalytic amount of piperidine (5%) was added dropwise and the reaction was stirred at refluxed for 4 h. The precipitated was filtered and the solid obtained was recrystallized in dichlomethane/methanol in a 73% yield. Mp 257–259 °C.

### Refinement

H atoms were treated as riding atoms with C—H(aromatic), 0.95Å with *U*_iso_ = 1.2U_eq_(C), C—H(methyl) = 0.98Å, with *U*_iso_ = 1.5U_eq_(C). The positions of methyl hydrogens were checked on a final difference map. The structure was refined as a two-component non-merohedral twin with a BASF parameter of 0.4374.

### Crystal data

**Table:**

C_17_H_11_NO_6_	*F*(000) = 672
*M**_r_* = 325.27	*D*_x_ = 1.544 Mg m^−^^3^
Monoclinic, *P*2_1_/*n*	Mo *K*α radiation, λ = 0.7107 Å
*a* = 8.875 (3) Å	Cell parameters from 1848 reflections
*b* = 17.266 (5) Å	θ = 2.4–26.2°
*c* = 9.174 (3) Å	µ = 0.12 mm^−^^1^
β = 95.401 (15)°	*T* = 100 K
*V* = 1399.6 (7) Å^3^	Prism, colourless
*Z* = 4	0.67 × 0.14 × 0.03 mm

### Data collection

**Table:**

Bruker APEXII CCD diffractometer	2864 independent reflections
Radiation source: fine-focus sealed tube	2200 reflections with *I* > 2σ(*I*)
Graphite monochromator	*R*_int_ = 0.054
ω and phi scans	θ_max_ = 26.5°, θ_min_ = 2.4°
Absorption correction: multi-scan (*SADABS*; Bruker, 2007)	*h* = −11→11
*T*_min_ = 0.604, *T*_max_ = 0.745	*k* = 0→21
30736 measured reflections	*l* = 0→11

### Refinement

**Table:**

Refinement on *F*^2^	Primary atom site location: structure-invariant direct methods
Least-squares matrix: full	Secondary atom site location: difference Fourier map
*R*[*F*^2^ > 2σ(*F*^2^)] = 0.043	Hydrogen site location: inferred from neighbouring sites
*wR*(*F*^2^) = 0.126	H-atom parameters constrained
*S* = 0.91	*w* = 1/[σ^2^(*F*_o_^2^) + (0.0871*P*)^2^ + 0.3903*P*] where *P* = (*F*_o_^2^ + 2*F*_c_^2^)/3
2864 reflections	(Δ/σ)_max_ < 0.001
219 parameters	Δρ_max_ = 0.39 e Å^−^^3^
0 restraints	Δρ_min_ = −0.26 e Å^−^^3^

### Special details

**Table:**

Experimental. ^1^H NMR (250 MHz, DMSO-*d_6_*) *δ* p.p.m. 8.29 (d, *J* = 3.2 Hz, 1H, H-4), 7.75 (dd, *J* = 9.8, 3.1 Hz, 1H, H-7), 7.69–7.56 (m, 3H, H-5, *o*-H-2, *o*-H-6), 7.04 (d, *J* = 8.3 Hz, 2H, *m*-H-3, *m*-H5), 6.08 (d, *J* = 9.6 Hz, 1H, H-8), 3.83 (s, 3H, –OMe); ^13^C NMR (63 MHz, DMSO-*d_6_*) *δ* p.p.m. 192.91, 177.98, 166.65, 162.34, 138.75, 131.40, 130.83, 130.47, 129.32, 128.05, 127.28, 121.24, 120.67, 113.84, 55.61; MS EI *m/z* (%): 326 ([*M*+1]+, 25), 325 ([*M*]+, 93), 190 (34), 135 (100), 92 (27), 77 (37); Elem. Anal. Calcd. for C_17_H_11_NO_6_: C, C, 62.77; H, 3.41; Found: C, 62.72; H, 3.32.
Geometry. All s.u.'s (except the s.u. in the dihedral angle between two l.s. planes) are estimated using the full covariance matrix. The cell s.u.'s are taken into account individually in the estimation of s.u.'s in distances, angles and torsion angles; correlations between s.u.'s in cell parameters are only used when they are defined by crystal symmetry. An approximate (isotropic) treatment of cell s.u.'s is used for estimating s.u.'s involving l.s. planes.
Refinement. Refinement of *F*^2^ against ALL reflections. The weighted *R*-factor *wR* and goodness of fit *S* are based on *F*^2^, conventional *R*-factors *R* are based on *F*, with *F* set to zero for negative *F*^2^. The threshold expression of *F*^2^ > 2σ(*F*^2^) is used only for calculating *R*-factors(gt) *etc*. and is not relevant to the choice of reflections for refinement. *R*-factors based on *F*^2^ are statistically about twice as large as those based on *F*, and *R*- factors based on ALL data will be even larger.

### Fractional atomic coordinates and isotropic or equivalent isotropic displacement parameters (Å^2^)

**Table:**

	*x*	*y*	*z*	*U*_iso_*/*U*_eq_	
C2	0.0307 (2)	0.13803 (12)	0.5978 (2)	0.0137 (4)	
C3	−0.0864 (2)	0.16362 (12)	0.6744 (2)	0.0153 (5)	
H3	−0.1256	0.2145	0.6595	0.018*	
C4	−0.1455 (2)	0.11473 (12)	0.7723 (2)	0.0164 (5)	
H4	−0.2265	0.1310	0.8257	0.020*	
C5	−0.0842 (2)	0.04053 (13)	0.7918 (2)	0.0156 (5)	
C6	0.0352 (2)	0.01480 (13)	0.7193 (2)	0.0147 (5)	
H6	0.0751	−0.0358	0.7364	0.018*	
C7	0.0969 (2)	0.06452 (12)	0.6200 (2)	0.0131 (4)	
C8	0.2244 (2)	0.04597 (12)	0.5416 (2)	0.0135 (4)	
H8	0.2701	−0.0036	0.5544	0.016*	
C9	0.2809 (2)	0.09697 (11)	0.4506 (2)	0.0131 (4)	
C10	0.2034 (2)	0.17126 (12)	0.4186 (2)	0.0149 (5)	
C15	0.4152 (2)	0.07478 (12)	0.3707 (2)	0.0146 (5)	
C17	0.5336 (2)	0.13255 (12)	0.3473 (2)	0.0147 (5)	
C18	0.5631 (2)	0.19667 (12)	0.4378 (2)	0.0144 (5)	
H18	0.5055	0.2041	0.5189	0.017*	
C19	0.6746 (2)	0.24980 (13)	0.4121 (2)	0.0154 (5)	
H19	0.6941	0.2928	0.4756	0.018*	
C20	0.7579 (2)	0.23946 (12)	0.2922 (2)	0.0154 (5)	
C21	0.7319 (2)	0.17463 (12)	0.2022 (2)	0.0171 (5)	
H21	0.7901	0.1671	0.1216	0.021*	
C22	0.6222 (2)	0.12166 (12)	0.2302 (2)	0.0146 (5)	
H22	0.6064	0.0773	0.1694	0.018*	
C24	0.9071 (3)	0.35368 (12)	0.3471 (2)	0.0205 (5)	
H24A	0.9528	0.3331	0.4406	0.031*	
H24B	0.8180	0.3847	0.3641	0.031*	
H24C	0.9809	0.3862	0.3027	0.031*	
N11	−0.1513 (2)	−0.01184 (11)	0.89252 (19)	0.0186 (4)	
O1	0.08130 (16)	0.18773 (8)	0.49739 (15)	0.0152 (3)	
O12	−0.11434 (19)	−0.08064 (9)	0.89156 (18)	0.0273 (4)	
O13	−0.24056 (19)	0.01484 (9)	0.97419 (17)	0.0248 (4)	
O14	0.23187 (18)	0.21826 (9)	0.32929 (17)	0.0224 (4)	
O16	0.42438 (17)	0.00770 (9)	0.32955 (17)	0.0196 (4)	
O23	0.86233 (17)	0.29040 (8)	0.24994 (16)	0.0185 (4)	

### Atomic displacement parameters (Å^2^)

**Table:**

	*U*^11^	*U*^22^	*U*^33^	*U*^12^	*U*^13^	*U*^23^
C2	0.0131 (10)	0.0141 (10)	0.0138 (10)	−0.0044 (9)	0.0010 (8)	0.0002 (8)
C3	0.0147 (11)	0.0139 (11)	0.0172 (11)	0.0022 (9)	0.0008 (9)	−0.0025 (8)
C4	0.0141 (11)	0.0192 (11)	0.0163 (11)	−0.0001 (9)	0.0030 (9)	−0.0046 (9)
C5	0.0140 (10)	0.0202 (11)	0.0127 (10)	−0.0056 (9)	0.0020 (8)	−0.0014 (9)
C6	0.0145 (11)	0.0150 (11)	0.0143 (11)	−0.0009 (9)	0.0004 (9)	−0.0011 (8)
C7	0.0102 (10)	0.0157 (10)	0.0132 (10)	−0.0015 (9)	0.0003 (8)	−0.0033 (8)
C8	0.0139 (10)	0.0110 (10)	0.0154 (10)	0.0009 (9)	−0.0006 (8)	−0.0031 (8)
C9	0.0120 (10)	0.0137 (11)	0.0140 (10)	−0.0018 (9)	0.0023 (8)	−0.0032 (8)
C10	0.0112 (11)	0.0165 (11)	0.0172 (11)	−0.0016 (9)	0.0034 (9)	−0.0018 (9)
C15	0.0134 (11)	0.0176 (12)	0.0130 (10)	0.0009 (9)	0.0017 (8)	0.0011 (8)
C17	0.0132 (11)	0.0170 (12)	0.0143 (10)	0.0037 (9)	0.0038 (8)	0.0031 (8)
C18	0.0120 (11)	0.0180 (12)	0.0136 (10)	0.0028 (9)	0.0037 (8)	0.0011 (8)
C19	0.0155 (11)	0.0161 (10)	0.0145 (11)	0.0022 (9)	0.0014 (8)	−0.0008 (9)
C20	0.0112 (10)	0.0180 (12)	0.0171 (11)	0.0019 (9)	0.0020 (8)	0.0054 (9)
C21	0.0154 (11)	0.0212 (12)	0.0155 (11)	0.0034 (9)	0.0063 (8)	0.0008 (9)
C22	0.0163 (11)	0.0129 (11)	0.0149 (10)	0.0034 (9)	0.0020 (8)	−0.0015 (8)
C24	0.0198 (12)	0.0183 (11)	0.0232 (12)	−0.0040 (10)	0.0013 (9)	0.0031 (9)
N11	0.0188 (10)	0.0222 (11)	0.0151 (10)	−0.0038 (8)	0.0033 (8)	−0.0001 (8)
O1	0.0138 (8)	0.0141 (7)	0.0183 (8)	0.0015 (6)	0.0047 (6)	0.0019 (6)
O12	0.0319 (10)	0.0213 (9)	0.0304 (10)	−0.0009 (7)	0.0119 (7)	0.0048 (7)
O13	0.0268 (9)	0.0303 (9)	0.0195 (8)	−0.0015 (8)	0.0134 (7)	−0.0017 (7)
O14	0.0203 (9)	0.0209 (8)	0.0271 (9)	0.0003 (7)	0.0076 (7)	0.0086 (7)
O16	0.0204 (8)	0.0160 (8)	0.0234 (9)	0.0007 (7)	0.0070 (7)	−0.0037 (6)
O23	0.0174 (8)	0.0188 (8)	0.0200 (8)	−0.0043 (7)	0.0060 (6)	0.0018 (6)

### Geometric parameters (Å, º)

**Table:**

C2—O1	1.365 (2)	C15—C17	1.479 (3)
C2—C3	1.381 (3)	C17—C18	1.394 (3)
C2—C7	1.405 (3)	C17—C22	1.403 (3)
C3—C4	1.372 (3)	C18—C19	1.386 (3)
C3—H3	0.9500	C18—H18	0.9500
C4—C5	1.397 (3)	C19—C20	1.393 (3)
C4—H4	0.9500	C19—H19	0.9500
C5—C6	1.377 (3)	C20—O23	1.361 (3)
C5—N11	1.459 (3)	C20—C21	1.397 (3)
C6—C7	1.400 (3)	C21—C22	1.377 (3)
C6—H6	0.9500	C21—H21	0.9500
C7—C8	1.433 (3)	C22—H22	0.9500
C8—C9	1.343 (3)	C24—O23	1.442 (3)
C8—H8	0.9500	C24—H24A	0.9800
C9—C10	1.472 (3)	C24—H24B	0.9800
C9—C15	1.506 (3)	C24—H24C	0.9800
C10—O14	1.197 (3)	N11—O13	1.230 (2)
C10—O1	1.387 (3)	N11—O12	1.233 (2)
C15—O16	1.223 (3)		
			
O1—C2—C3	116.95 (18)	C18—C17—C22	118.4 (2)
O1—C2—C7	120.40 (19)	C18—C17—C15	123.04 (19)
C3—C2—C7	122.65 (19)	C22—C17—C15	118.55 (19)
C4—C3—C2	119.25 (19)	C19—C18—C17	121.4 (2)
C4—C3—H3	120.4	C19—C18—H18	119.3
C2—C3—H3	120.4	C17—C18—H18	119.3
C3—C4—C5	118.7 (2)	C18—C19—C20	119.3 (2)
C3—C4—H4	120.7	C18—C19—H19	120.3
C5—C4—H4	120.7	C20—C19—H19	120.3
C6—C5—C4	122.8 (2)	O23—C20—C19	124.6 (2)
C6—C5—N11	118.98 (19)	O23—C20—C21	115.35 (19)
C4—C5—N11	118.18 (19)	C19—C20—C21	120.0 (2)
C5—C6—C7	118.8 (2)	C22—C21—C20	120.1 (2)
C5—C6—H6	120.6	C22—C21—H21	119.9
C7—C6—H6	120.6	C20—C21—H21	119.9
C6—C7—C2	117.68 (19)	C21—C22—C17	120.7 (2)
C6—C7—C8	124.40 (19)	C21—C22—H22	119.6
C2—C7—C8	117.91 (19)	C17—C22—H22	119.6
C9—C8—C7	121.61 (19)	O23—C24—H24A	109.5
C9—C8—H8	119.2	O23—C24—H24B	109.5
C7—C8—H8	119.2	H24A—C24—H24B	109.5
C8—C9—C10	120.03 (19)	O23—C24—H24C	109.5
C8—C9—C15	119.64 (18)	H24A—C24—H24C	109.5
C10—C9—C15	120.09 (18)	H24B—C24—H24C	109.5
O14—C10—O1	116.33 (19)	O13—N11—O12	123.53 (18)
O14—C10—C9	127.0 (2)	O13—N11—C5	118.57 (18)
O1—C10—C9	116.64 (18)	O12—N11—C5	117.90 (18)
O16—C15—C17	121.65 (19)	C2—O1—C10	123.03 (16)
O16—C15—C9	118.01 (19)	C20—O23—C24	117.96 (17)
C17—C15—C9	120.31 (18)		
			
O1—C2—C3—C4	177.38 (18)	O16—C15—C17—C18	−152.9 (2)
C7—C2—C3—C4	−2.6 (3)	C9—C15—C17—C18	25.3 (3)
C2—C3—C4—C5	0.4 (3)	O16—C15—C17—C22	25.9 (3)
C3—C4—C5—C6	1.4 (3)	C9—C15—C17—C22	−155.83 (19)
C3—C4—C5—N11	−177.98 (19)	C22—C17—C18—C19	1.4 (3)
C4—C5—C6—C7	−1.0 (3)	C15—C17—C18—C19	−179.72 (19)
N11—C5—C6—C7	178.44 (18)	C17—C18—C19—C20	0.8 (3)
C5—C6—C7—C2	−1.2 (3)	C18—C19—C20—O23	175.13 (19)
C5—C6—C7—C8	177.66 (19)	C18—C19—C20—C21	−2.1 (3)
O1—C2—C7—C6	−176.97 (17)	O23—C20—C21—C22	−176.26 (18)
C3—C2—C7—C6	3.1 (3)	C19—C20—C21—C22	1.2 (3)
O1—C2—C7—C8	4.1 (3)	C20—C21—C22—C17	1.0 (3)
C3—C2—C7—C8	−175.89 (19)	C18—C17—C22—C21	−2.3 (3)
C6—C7—C8—C9	−178.2 (2)	C15—C17—C22—C21	178.75 (19)
C2—C7—C8—C9	0.6 (3)	C6—C5—N11—O13	168.4 (2)
C7—C8—C9—C10	−5.7 (3)	C4—C5—N11—O13	−12.2 (3)
C7—C8—C9—C15	179.94 (18)	C6—C5—N11—O12	−11.2 (3)
C8—C9—C10—O14	−171.9 (2)	C4—C5—N11—O12	168.27 (19)
C15—C9—C10—O14	2.5 (3)	C3—C2—O1—C10	176.38 (18)
C8—C9—C10—O1	6.1 (3)	C7—C2—O1—C10	−3.6 (3)
C15—C9—C10—O1	−179.58 (17)	O14—C10—O1—C2	176.75 (18)
C8—C9—C15—O16	35.9 (3)	C9—C10—O1—C2	−1.4 (3)
C10—C9—C15—O16	−138.5 (2)	C19—C20—O23—C24	9.7 (3)
C8—C9—C15—C17	−142.4 (2)	C21—C20—O23—C24	−172.89 (18)
C10—C9—C15—C17	43.2 (3)		

## References

[bb1] Altomare, A., Burla, M. C., Camalli, M., Cascarano, G. L., Giacovazzo, C., Guagliardi, A., Moliterni, A. G. G., Polidori, G. & Spagna, R. (1999). *J. Appl. Cryst.* **32**, 115–119.

[bb2] Borges, F., Roleira, F., Milhazes, N., Uriarte, E. & Santana, L. (2009). *Front. Med. Chem.* **4**, 23–85.

[bb3] Bruker (2007). *APEX2*, *SAINT* and *SADABS* Bruker AXS Inc., Madison, Wisconsin, USA.

[bb4] Farrugia, L. J. (2012). *J. Appl. Cryst.* **45**, 849–854.

[bb5] Matos, M. J., Santana, L., Uriarte, E., Delogu, G., Corda, M., Fadda, M. B., Era, B. & Fais, A. (2011*c*). *Bioorg. Med. Chem. Lett.* **21**, 3342–3345. 10.1016/j.bmcl.2011.04.012 21514152

[bb6] Matos, M. J., Terán, C., Pérez-Castillo, Y., Uriarte, E., Santana, L. & Viña, D. (2011*a*). *J. Med. Chem.* **54**, 7127–7137. 10.1021/jm200716y 21923181

[bb7] Matos, M. J., Vazquez-Rodriguez, S., Santan, L., Uriarte, E. & Viña, D. (2011*b*). *Bioorg. Med. Chem. Lett.* **21**, 4224–4227. 10.1016/j.bmcl.2011.05.074 21684743

[bb8] Raju, B. C., Tiwari, A. K., Kumar, J. A., Ali, A. Z., Agawane, S. B., Saidachary, G. & Madhusudana, K. (2010). *Bioorg. Med. Chem.* **18**, 358–365. 10.1016/j.bmc.2009.10.047 19932027

[bb9] Sheldrick, G. M. (2008). *Acta Cryst.* A**64**, 112–122. 10.1107/S0108767307043930 18156677

[bb10] Vazquez-Rodriguez, S., Matos, M. J., Santana, L., Uriarte, E., Borges, F., Kachler, S. & Klotz, K.-N. (2013). *J. Pharm. Pharmacol.* DOI: 10.1111/jphp.12028. 10.1111/jphp.12028 23600387

[bb11] Viña, D., Matos, M. J., Ferino, G., Cadoni, E., Laguna, R., Borges, F., Uriarte, E. & Santana, L. (2012*b*). *Chem. Med. Chem.* **7**, 464–470. 10.1002/cmdc.201100538 22287164

[bb12] Viña, D., Matos, M. J., Yáñez, M., Santana, L. & Uriarte, E. (2012*a*). *Med. Chem. Commun.* **3**, 213–218.

